# New Advances in the Study of CMTM6, a Focus on Its Novel Non-Canonical Cellular Locations, and Functions beyond Its Role as a PD-L1 Stabilizer

**DOI:** 10.3390/cancers16183126

**Published:** 2024-09-11

**Authors:** Pedro Ivan Urciaga-Gutierrez, Ramon Antonio Franco-Topete, Blanca Estela Bastidas-Ramirez, Fabiola Solorzano-Ibarra, Jose Manuel Rojas-Diaz, Nadia Tatiana Garcia-Barrientos, Ksenia Klimov-Kravtchenko, Martha Cecilia Tellez-Bañuelos, Pablo Cesar Ortiz-Lazareno, Oscar Peralta-Zaragoza, Angelica Meneses-Acosta, Alan Guillermo Alejandre-Gonzalez, Miriam Ruth Bueno-Topete, Jesse Haramati, Susana del Toro-Arreola

**Affiliations:** 1 Instituto de Investigación en Enfermedades Crónico Degenerativas, Departamento de Biología Molecular y Genómica, Centro Universitario de Ciencias de la Salud, Universidad de Guadalajara, Guadalajara 44340, Jalisco, Mexiconadia.garcia2322@alumnos.udg.mx (N.T.G.-B.);; 2 Laboratorio de Patología, Departamento de Microbiología y Patología, Centro Universitario de Ciencias de la Salud, Universidad de Guadalajara, Guadalajara 44340, Jalisco, Mexico; 3 Laboratorio de Inmunología Traslacional, Departamento de Biología Celular y Molecular, Centro Universitario de Ciencias Biológicas y Agropecuarias, Universidad de Guadalajara, Zapopan 45180, Jalisco, Mexico; 4 División de Inmunología, Centro de Investigación Biomédica de Occidente, Instituto Mexicano del Seguro Social, Guadalajara 44716, Jalisco, Mexico; 5 Dirección de Infecciones Crónicas y Cáncer, Centro de Investigación sobre Enfermedades Infecciosas, Instituto Nacional de Salud Pública, Cuernavaca 62100, Morelos, Mexico; 6 Laboratorio de Biotecnología Farmacéutica, Facultad de Farmacia, Universidad Autónoma del Estado de Morelos, Cuernavaca 62209, Morelos, Mexico; 7 Laboratorio de Inmunología, Departamento de Fisiología, Centro Universitario de Ciencias de la Salud, Universidad de Guadalajara, Guadalajara 44340, Jalisco, Mexico

**Keywords:** CMTM6, PD-1, PD-L1, cancer, immunotherapy

## Abstract

**Simple Summary:**

Recently, CMTM6 has been found to stabilize PD-L1 on the plasma membrane of some tumor cells, favoring tumor immune system evasion. Until the present, it was unknown whether this protein could be present in a soluble form in the plasma of patients with cervical cancer (CC) or, additionally, if it could be found in atypical subcellular compartments in cell lines derived from CC. The results of our present research confirm that CMTM6 is increased in the plasma of patients with CC and associated, but not exclusively so, with exosomes, and that exosomal CMTM6 levels are correlated with exosomal PD-L1 levels. In addition, in CC cell lines, CMTM6 was found to be secreted and present both on the membrane and intracellularly. CMTM6 derived from CC in soluble, exosomal membrane and intracellular forms could have an impact on the biology of tumor cells by affecting the expression of molecules associated with tumor development and progression, such as PD-L1.

**Abstract:**

CMTM6 is a membrane protein that acts as a regulator of PD-L1, maintaining its expression on the cell surface, and can prevent its lysosome-mediated degradation. It is unknown if CMTM6 is present in the plasma of patients with cervical cancer, and if it has non-canonical subcellular localizations in cell lines derived from cervical cancer. Our objective was to determine whether CMTM6 is found in plasma derived from cervical cancer patients and its subcellular localization in cell lines. Patient plasma was separated into exosome-enriched, exosome-free, and total plasma fractions. The levels of CMTM6 in each fraction were determined using ELISA and Western blot. Finally, for the cellular model, HeLa, SiHa, CaSki, and HaCaT were used; the subcellular locations of CMTM6 were determined using immunofluorescence and flow cytometry. Soluble CMTM6 was found to be elevated in plasma from patients with cervical cancer, with a nearly three-fold increase in patients (966.27 pg/mL in patients vs. 363.54 pg/mL in controls). CMTM6 was preferentially, but not exclusively, found in the exosome-enriched plasma fraction, and was positively correlated with exosomal PD-L1; CMTM6 was identified in the membrane, intracellular compartments, and culture supernatant of the cell lines. These results highlight that CMTM6, in its various presentations, may play an important role in the biology of tumor cells and in immune system evasion.

## 1. Introduction

The immune system plays an indispensable role in fighting and clearing abnormal cells, including tumor cells. These cells can evade and resist killing mediated by the immune system through different mechanisms. One such mechanism is increasing the expression of immune checkpoint proteins such as programmed cell death-1 (PD-1) and its ligand programmed cell death-ligand 1 (PD-L1) [1]. PD-1 is an immune checkpoint receptor expressed on the surface of many types of immune cells. PD-L1 is a member of the B7 family that is expressed in non-lymphoid organs, antigen-presenting cells (APCs), and nonhematopoietic cells. Under physiological conditions, PD-1 interacts with PD-L1, and this provides inhibitory signals to promote the maintenance of self-tolerance. However, aberrant expression of PD-L1 in several types of tumors renders T cells inactive or nonfunctional [2]. PD-L1, a protein that is capable of strongly modulating the adaptive arm of the immune system, is highly inducible by cytokine stimuli such as IFN-γ, or pathological conditions such as the presence of viral or cancer-associated antigens [3]. Recent studies have demonstrated that patients with active human papillomavirus (HPV) infection show high expression of PD-L1 in dysplastic squamous cells, typically towards the base of cervical neoplasias, as well as in malignant squamous cells of cervical cancer. This expression has been strongly associated with a possible “crosstalk” leading to the induction of PD-1 on CD8 T cells that are recruited to the area of viral infection [4,5]. While the membrane form of PD-L1 has been the most studied in cervical cancer (CC), this molecule has also been reported to be released in a soluble form and to be found in other subcellular fractions, such as extracellular vesicles (EVs) [6,7,8]. The above suggests that this molecule has additional roles, not just those ascribed to cell–cell interaction due to its membrane-bound form. 

In recent years, it has been proposed that one of the main master regulators of PD-L1 expression in some tumors is the CMTM protein family. This family consists of nine members (CMTM1–CMTM8 and CKLF); several members of this family are either overexpressed or inhibited in various tumors, such as pancreatic and gastric cancer, which affect cell proliferation and patient survival, suggesting their involvement in tumorigenesis and their prognostic value [9]. An important member of this family that is relevant due to its strong association with PD-L1 is CMTM6 (CKLF-Like MARVEL Transmembrane Domain Containing 6). This protein contains a MARVEL (MAL and related proteins for vesicle trafficking and membrane link) region as well as four transmembrane structures and plays a key role in the trafficking of transmembrane proteins and secretory proteins [10]. Previously considered an orphan transmembrane molecule, of late CMTM6 has been reported to bind strongly to PD-L1 and stabilize it on the plasma membrane, resulting in a greater inhibitory impact on the immune system and successful escape from immune surveillance [11]. In the context of the CMTM6/PD-L1 axis, CMTM6 co-localizes with PD-L1 and prevents PD-L1 from becoming a target for lysosome-mediated degradation. CMTM6 also promotes the transport of PD-L1 to the recycling endosome, leading to a decrease in delivery to the late endosome and lysosome, as well as reducing PD-L1 ubiquitination [9]. In recent years, CMTM6 has additionally been associated with M2 macrophage polarization, tumor proliferation, metastasis, and reduced survival time [12,13,14]. And although little is known about the subcellular location of CMTM6, as it is highly associated with PD-L1, it is known that it is found in the plasma membrane, although recent reports indicate that it is also present in tissues such as lungs, breasts, liver, skin, and cervix [15,16,17]. And, interestingly, in addition to being reported on the plasma membrane of tissues, CMTM6 was described to be present in exosomes [12]. However, although normal expression of CMTM6 is known to occur in the cervical cavity, it is not known if this protein is found in the plasma of patients with CC, and, more importantly, if it is present in a soluble form or associated with exosomes. Therefore, our main objective was to elucidate the presence of soluble CMTM6 (sCMTM6) in CC using plasma fractions from patients (total plasma, exosome-free, and exosome-enriched) and healthy donors (HDs) to determine if sCMTM6 levels are increased and, if so, in what serum fraction they are found. Additionally, we used CC cell line models to validate if this molecule is present in other non-typical cellular sub-locations to explore the possible role that sCMTM6 might have in immune evasion.

## 2. Materials and Methods

### 2.1. Sample Collection and Preparation

We utilized 23 samples in the CC group and 23 age-matched control samples in the HDs group for analysis of CMTM6. For the analysis of PD-L1, we utilized 21 samples in the CC group and 11 in the HDs group. Participant data are shown in Table 1 and Table 2.

The participants in the CC groups were women recruited from the Antiguo Hospital Civil de Guadalajara Fray Antonio Alcalde and the Instituto Jalisciense de Cancerología. Age-matched clinically healthy donors (HDs) formed the control group. The study was conducted following the ethical principles stated in the Declaration of Helsinki. The approval by the institutional research, bioethics, and biosecurity committees was obtained under the number CI-02522 and all study participants gave their written informed consent. Peripheral blood (5 mL) was collected by venipuncture using tubes with anticoagulant, BD Vacutainer K2 EDTA (Becton, Dickinson and Company, Franklin Lakes, NJ, USA). The blood was allowed to stand for 30 min, and then the blood was centrifuged at 1500 rpm for 15 min at room temperature. The plasma was then separated into tubes and stored at −80°.

### 2.2. Cell Culture

Cell lines SiHa, HeLa, CaSki, and HaCaT were grown as monolayer cultures in Dulbecco’s modified Eagle’s medium (Invitrogen, Waltham, MA, USA) containing 10% Exosome-Depleted Fetal Bovine Serum (Invitrogen) and antibiotics (100 units/mL penicillin and 100 μg/mL streptomycin) in a humidified atmosphere of 5% CO_2_ at 37 °C.

### 2.3. Isolation of Exosomes and Western Blot

Exosome isolation was performed with the Total Exosome Isolation Reagent (Cat. 4478360, Invitrogen) according to the manufacturer’s instructions. After defrosting, samples were centrifuged at 2000× *g*/30 min to remove any cellular debris. The isolation reagent (20–100 μL) was then added to the debris-free plasma (at a ratio of 20 μL reagent/100 μL plasma), which was subsequently vortexed and then incubated for 30 min at 2 °C to 8 °C. The samples were next centrifuged at 12,000× *g*/10 min and the exosome pellet was obtained. The amount of protein in each sample was determined by spectrophotometry with NanoDrop One (ThermoFisher Scientific, Waltham, MA, USA). The samples (denatured with DTT at 95 °C for 5 min, 170 µg/lane) were resolved and analyzed by SDS-PAGE and immunoblotting using the CMTM6 antibody (anti-CMTM6, Cat. 19130S, Cell Signaling Technology, Danvers, MA, USA); furthermore, the vesicle marker of exosomes (anti-CD63, Cat. sc-5275, lot number C1317, Santa Cruz Biotechnology, Dallas, TX, USA) was used to verify the purity of our separated serum fractions. Primary antibody binding was detected using HRP Donkey anti-Rabbit IgG (Cat. 406401, m-IgGκ BP-HRP: sc-516102, Santa Cruz Biotechnology). Densitometric analysis was performed using VisionWorks V11.2 software (Analytik Jena, Jena, Germany) and data were normalized against the β-actin signal in each lane.

### 2.4. ELISA

Soluble PD-L1 and CMTM6 concentrations in the samples (CC patients and healthy donor plasma and cell line-derived supernatants) were measured according to the manufacturer’s protocols. Human PD-L1 ELISA Kit, Lot 356381-001, REF BMS2327 (ThermoFisher, Waltham, MA, USA) and HUMAN CMTM6 ELISA Kit (Colorimetric), CAT NBP2-75298 (NOVUS BIOLOGICALS, Centennial, CO, USA) were used. Briefly, samples in duplicate were added to the sample wells. Standards and samples were added to each well and incubated for 90 min at 37 °C; after this time, the liquid was removed, the wells washed, biotinylated detection Ab working solution added, and incubated for 1 h at the same temperature. Next, wells were washed 4× with wash buffer and then incubated with HRP conjugate working solution for 30 min at 37 °C. Wells were then washed 4×, then incubated for 15 min at the same temperature with the substrate reagent before stopping the reaction with the Stop Solution and reading the absorbance at 450 nm.

### 2.5. Immunofluorescence

To identify the cell locations of CMTM6 and PD-L1, an immunofluorescence experiment was performed according to the following protocol. A total of 20,000 SiHa, HeLa, HaCaT, and CaSki cells were seeded in an 8-well Glass Slide Nunc™ Lab-Tek™ Chamber Slide™, Cat. 177402 and incubated for 48 h to assure confluence and adherence, after which time they were fixed with 4% paraformaldehyde. They were then washed with PBS 3 times and subsequently permeabilized by incubating for 10 min at room temperature with 100 μL of 0.2% Tween. The washing with PBS was then repeated. Blocking was carried out for 30 min at room temperature with a blocking buffer that contained 1% FBS diluted in sterile PBS. After blocking, the cells were washed again with PBS 3 times. The corresponding dilutions were made and incubated with the primary antibodies overnight at 4 °C. For CMTM6, rabbit CMTM6 polyclonal antibody, Cat. PA5-55472, Thermo Fisher, 1:1000; for PD-L1, mouse PD-L1 monoclonal antibody [8E12], MyBiosource, Cat. MBS154571, 1:1000. Afterwards, washes were performed with PBS and the secondary antibodies (Alexa Fluor 488 anti-mouse IgG1 antibody, Biolegend (San Diego, CA, USA), Cat. 406626, 1:300 and goat anti-rabbit IgG H + L cross-adsorbed secondary antibody, Alexa Fluor 594, Invitrogen, Cat. A11012, 1:1000) were diluted and incubated with the cells for one hour at room temperature. The cells were washed again with PBS, incubated with 200 μL of DAPI 1:10,000 for 5 min, and finally washed with PBS. Images were captured using the ZOE Fluorescent Cell Imager from Bio-Rad, Hercules, CA, USA.

### 2.6. Flow Cytometry

A flow cytometry protocol was used to analyze the expression of PD-L1 and CMTM6. SiHa, HeLa, HaCaT, and CaSki cells were used for this portion of the study. Two types of flow cytometry experiments, membrane and intracellular, were performed. The following antibodies were used to stain 1 × 10^5^ cells: PE anti-human CD274, Clone 29E.2A3, Cat. 329706, Biolegend; APC Anti-CMTM6 antibody [EPR23015-45], Cat. ab306353, Abcam (Boston, MA, USA); and APC Rabbit isotype control antibody, Clone EPR25A, Cat. Ab232814, Abcam. Data acquisition was performed using an Attune NxT flow cytometer. For the acquisition of each sample, an initial dot plot (FSC-A vs. FSC-H) was first derived in order to identify singlets. This selection generated a dot plot with the combination FSC-A vs. SSC-A, after which 100,000 events were acquired from the singlet cell line gate. The results were analyzed with Kaluza software V2.1 (Beckman Coulter, Brea, CA, USA). Isotype controls were used to adjust background fluorescence. For intracellular flow cytometry, the Biolegend Cyto-Fast™ Fix/Perm Buffer Set Cat. 426803 protocol was used according to the manufacturer’s specifications. A separate aliquot of cells was used for the intracellular staining vs. the membrane staining. The same antibodies as specified above were used, with the exception being that for CMTM6, the same un-labeled antibody as used in the Western blot above (anti-CMTM6, Cat. 19130S, Cell Signaling) was used at 1:200, followed by the secondary antibody FITC Donkey anti-rabbit IgG (minimal x-reactivity), Cat. 406403, Biolegend, at 1:500. The cells used for intracellular staining were resuspended in 250 μL of Cytofax Fix/Perm solution and incubated for 20 min at room temperature; after incubation, the cells were washed twice with Cyto-Fast™ Perm Wash Solution and centrifuged at 1800 rpm for 5 min and the supernatant was decanted with a micropipette to conserve the pellet. Finally, cells were stained with the antibodies mentioned above using the same data acquisition cytometer.

### 2.7. Statistical Analysis

Data were assessed for normality using the Kolmogorov–Smirnov test and comparisons between the groups were performed using Student’s *t*-test, Wilcoxon test, ANOVA, or Pearson correlation. A *p*-value < 0.05 was considered statistically significant. GraphPad Prism version 9 software was used for these analyses. Only significant *p*-values are displayed.

## 3. Results

### 3.1. Plasma CMTM6 and PD-L1 Are Increased in Cervical Cancer

**Figure 1:**
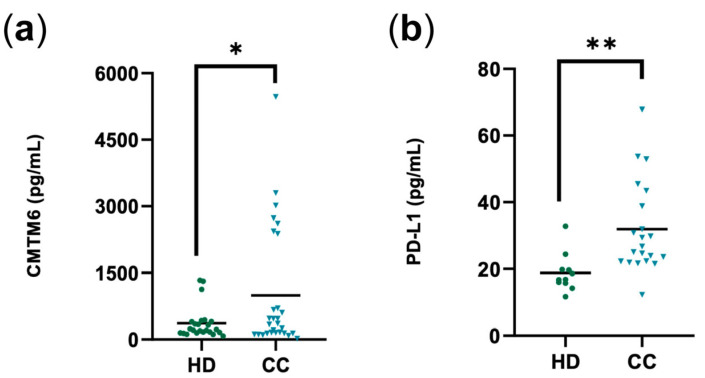
Total CMTM6 and total PD-L1 in plasma from the healthy donors (HDs) group and the cervical cancer patients (CC) group. (**a**) Concentrations of CMTM6 in the plasma of HDs (*n* = 23) and CC (*n* = 23) were measured by enzyme-linked immunosorbent assay (ELISA). Data are shown as pg/mL of CMTM6. (**b**) PD-L1 levels in the plasma of HDs (*n* = 11) and CC (*n* = 21) are shown as pg/mL of PD-L1. Student’s *t*-test was used. * *p* ≤ 0.05, ** *p* ≤ 0.01.

We performed an ELISA on plasma samples from HDs (*n* = 23) and CC patients (*n* = 23) to evaluate the plasma levels of CMTM6 (Figure 1). We observed a significant increase in the concentration of plasma CMTM6 in CC compared to HDs (Figure 1a). Of note was that the majority (12 of 23) of the CC patients had higher plasma CMTM6 levels (966.27 pg/mL) than the mean HD levels (363.54 pg/mL). We next performed an ELISA test to assess plasma PD-L1 levels. A significant increase was observed in the concentrations of plasma PD-L1 in CC compared to HDs (mean HDs 18.80 pg/mL and CC 31.93 pg/mL) (Figure 1b). Notably, all but one of the CC samples had higher plasma PD-L1 levels than the mean HD levels (18.80 pg/mL). These results suggest a relationship between the release of both proteins in cervical cancer, but it is unknown if these levels are being released in a soluble manner or confined to microvesicles (EVs) such as exosomes.

### 3.2. CMTM6 and PD-L1 Are Preferentially Released in Exosomes

**Figure 2:**
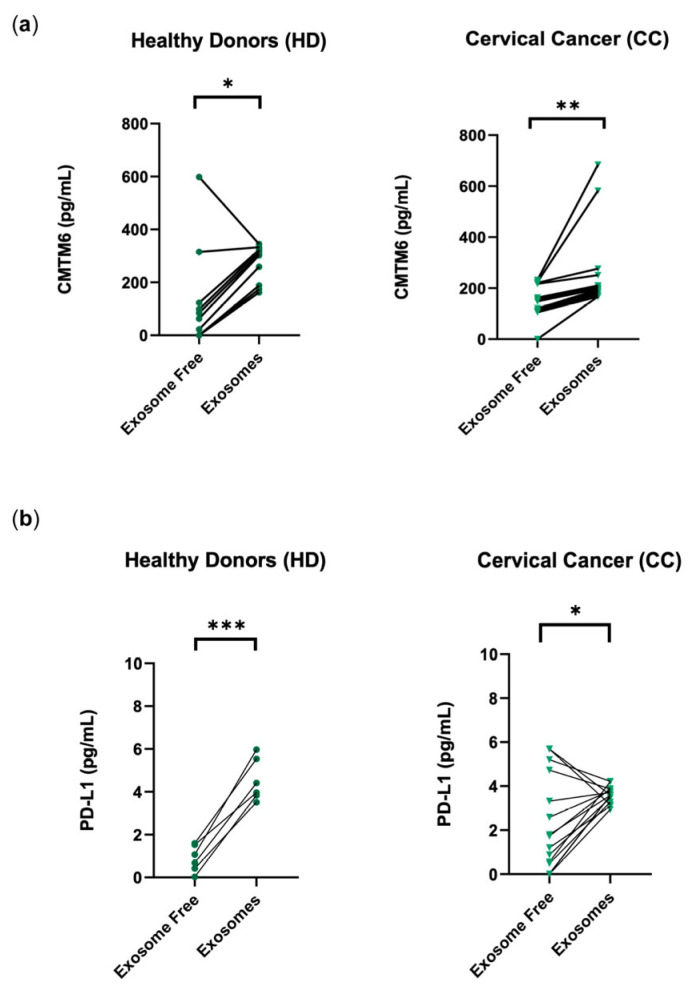

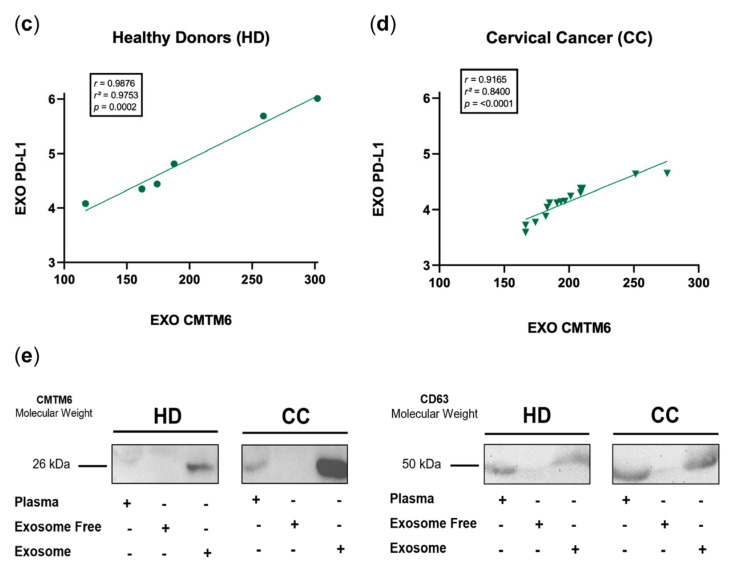
CMTM6 and PD-L1 were found in exosomes from the plasma of the healthy donor group (HD) and the group of patients with cervical cancer (CC). (**a**) CMTM6 is preferentially released in exosomes in both the HD (*n* = 10) and CC (*n* = 20) groups; data shown as pg/mL of CMTM6. (**b**) PD-L1 was elevated in the exosome-enriched plasma fractions from both the HDs (*n* = 6) and CC (*n* = 15); data shown as pg/mL. (**c**) Correlation between exosomal CMTM6 and PD-L1 in HD samples (*r* = 0.996). (**d**) Correlation between exosomal CMTM6 and PD-L1 in CC samples (*r* = 0.8346). (**e**) CMTM6 was found to increase in the exosomal fractions from HDs and CC patients by Western blot. CD63 was used as an exosomal marker. The uncropped blots are shown in Figure S1. Wilcoxon ranked test was used. * *p* ≤ 0.05, ** *p* ≤ 0.01, *** *p* < 0.005.

Once we observed that CMTM6 and PD-L1 were increased in the plasma of patients with cervical cancer, we decided to fraction the plasma into exosome-free and exosome-enriched fractions. We again performed an ELISA, but now using plasma fractions from healthy donors (*n* = 10) and fractions from patients with cervical cancer (*n* = 20) to determine the levels of CMTM6 per fraction (Figure 2). The exosomal CMTM6 levels in cervical cancer patients were non-significantly higher than the exosomal CMTM6 levels in HDs (349.59 in CC vs. 299.51 in HDs). Additionally, we observed that sCMTM6 is more likely to be found in the exosome-enriched, but not exosome-free, fractions from both patients and controls (Figure 2a).

To elucidate in which fraction the highest concentration of PD-L1 was released, we again decided to fraction the plasma into exosome-free and exosome-enriched fractions. We performed an ELISA, but now using the plasma fractions from healthy donors (*n* = 6) and patients (*n* = 15) to evaluate PD-L1 levels on a fraction-by-fraction basis (Figure 2b). Exosomal PD-L1 levels were significantly higher than exosome-free PD-L1 levels in both HDs and CC. Levels of HD exosomal CMTM6 and PD-L1 were significantly positively correlated with each other (*r* = 0.9876) (Figure 2c), and levels of CC exosomal CMTM6 and PD-L1 were significantly positively correlated with each other (*r* = 0.9165) (Figure 2d).

To verify these results, a Western blot analysis was carried out on the plasma fractions. An approximately 26 kDa band was identified corresponding to the molecular weight of CMTM6 and this band was found predominantly in the exosome fractions from both HDs and CC; however, the band appeared relatively denser in the CC sample. The marker CD63 (tetraspanin family member used as an exosome marker) was used to verify the corresponding exosomal fractions (Figure 2e).

### 3.3. CMTM6 and PD-L1 Are Present in CC-Derived Cell Line Lysates

**Figure 3:**
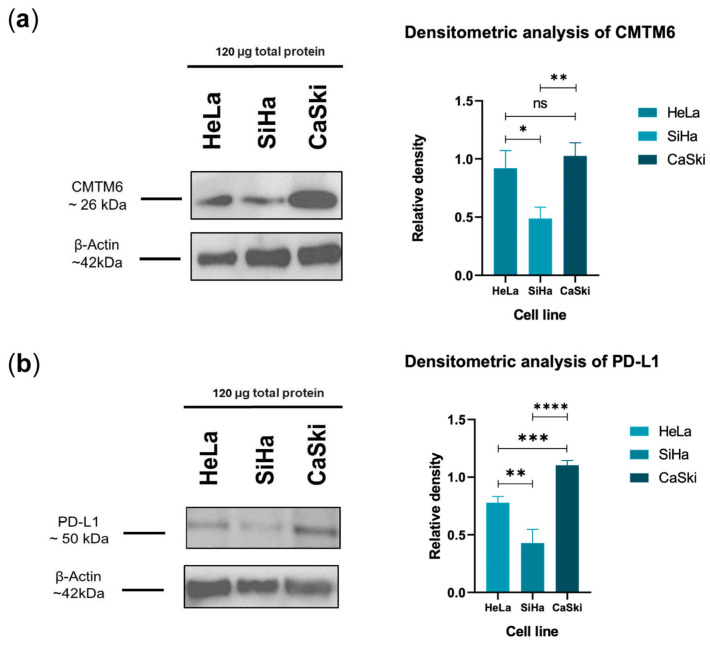
CMTM6 and PD-L1 were found in the lysates of CC-derived cells. (**a**) CMTM6 expression was found to be higher in the total lysate of CaSki cells compared to those of HeLa and SiHa cells, shown as bands corresponding to the approximate molecular weight reported for CMTM6 and its densitometric analysis. (**b**) The bands corresponding to the molecular weight reported for PD-L1 were also found in the three cell lines, coinciding again to show that CaSki cells express the highest levels of this protein; the band pattern and its densitometric analysis are shown. In both cases, β-actin was used as a constitutive protein and loading control. The uncropped blots are shown in Figure S2. Data are shown as mean ± SD; three independent experiments were performed for each condition. * *p* ≤ 0.05, ** *p* ≤ 0.01, *** *p* ≤ 0.001, **** *p* ≤ 0.0001, ns: no significance.

When we identified that sCMTM6 and sPD-L1 were present in the plasma of CC patients, we decided to try to elucidate whether these molecules could come from the tumor cells, so we designed a Western blot analysis using total protein from the lysates of CC-derived cell lines SiHa, HeLa, and CaSki (Figure 3). We identified in the three cell lines a band of approximately 26 kDa, which coincides with the molecular weight reported for CMTM6. Interestingly, the CaSki cell line (derived from metastasis) showed a relatively increased density and size of its bands compared to HeLa and SiHa cells (Figure 3a). In the case of PD-L1, when we analyzed the relative density and bands in the lysate of the cell lines derived from CC patients, the same phenomenon was observed: CaSki was the cell line with the highest expression of PD-L1 (Figure 3b). Both results are consistent with the idea that CMTM6 and PD-L1 are present together in different cervical cancer cell lines, as has been seen in other cancers and cell line models of cancers.

### 3.4. CMTM6 Found in the Cell Membrane and Intracellularly in Cell Lines Derived from Cervical Cancer

**Figure 4:**
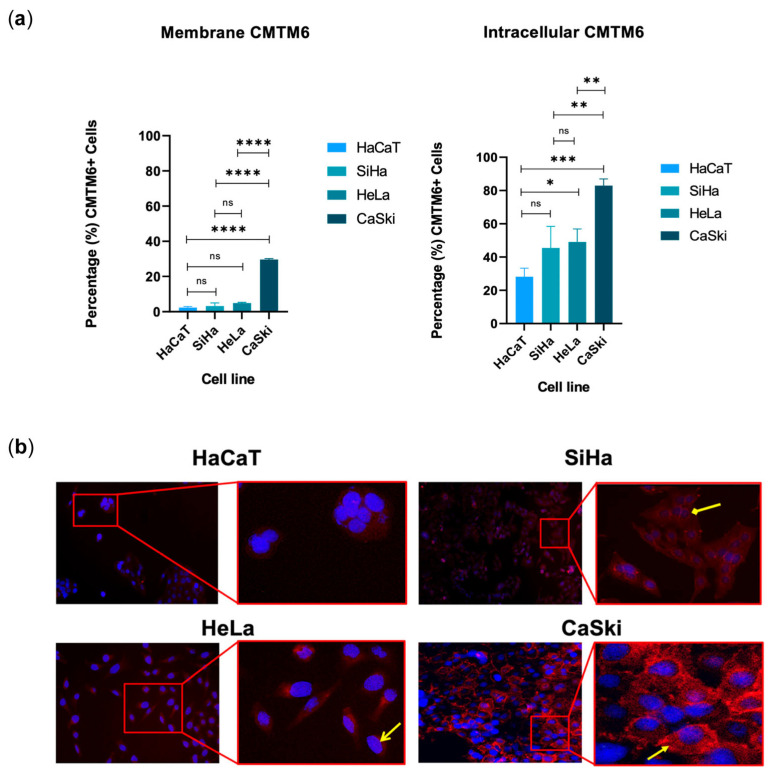
CMTM6 was found in the cell membrane and intracellularly in cell lines derived from cervical cancer. (**a**) The percentage of CMTM6-positive cells was determined by flow cytometry. CMTM6 was found both intracellularly and associated with the plasma membranes of all cell lines. Interestingly, the highest percentages of CMTM6, both intracellular and on the membrane, were found in CaSki cells. Data are shown as mean ± SD; three independent experiments were performed for each condition. * *p* ≤ 0.05, ** *p* ≤ 0.01, *** *p* ≤ 0.001, **** *p* ≤ 0.0001, ns: no significance. (**b**) Immunofluorescence staining verified that CMTM6 (AF-594, red stain) is found in different subcellular locations such as intracellular (cytoplasm shown with diamond-tipped arrow) and the plasma membrane (closed arrow). The nuclei (open arrow) are stained with DAPI (blue). Note the intracellular staining in SiHa cells, which obscures the nucleus, and the visible nucleus and membrane staining in CaSki cells. Images taken using the 10× objective (left side) and 30× (right side).

As CMTM6 was found in the total lysates of the cell lines derived from cervical cancer, flow cytometry (both intracellular and traditional membrane) was used to quantify the relative expression of CMTM6 in these two locations (Figure 4). For this, the SiHa, HeLa, and CaSki cell lines were used again; in addition, HaCaT cells (an immortalized human keratinocyte line) were used as a control. All cell lines expressed intracellular CMTM6 and membrane CMTM6 (Figure 4a), with CaSki expressing both the highest intracellular and membrane percentages. In all four cell lines, the intracellular staining was notably higher than the membrane staining; HaCat, HeLa, and SiHa cells exhibited very low membrane staining. It is also notable that the same order of staining was preserved between intracellular and membrane staining; that is to say, CaSki cells had the highest, followed by HeLa cells, SiHa cells, and then HaCat cells. To validate these results, an immunofluorescence experiment was designed, verifying that of the four cell lines, CaSki cells exhibited the most prominent membrane staining, while HeLa and SiHa cells exhibited intracellular but no observable membrane staining, and HaCat cells exhibited comparatively lower staining, both membrane and intracellular, compared with the other three tumorigenic cell lines (Figure 4b). 

### 3.5. PD-L1 Was Found in Its Typical Membrane-Associated Form in Cell Lines

**Figure 5:**
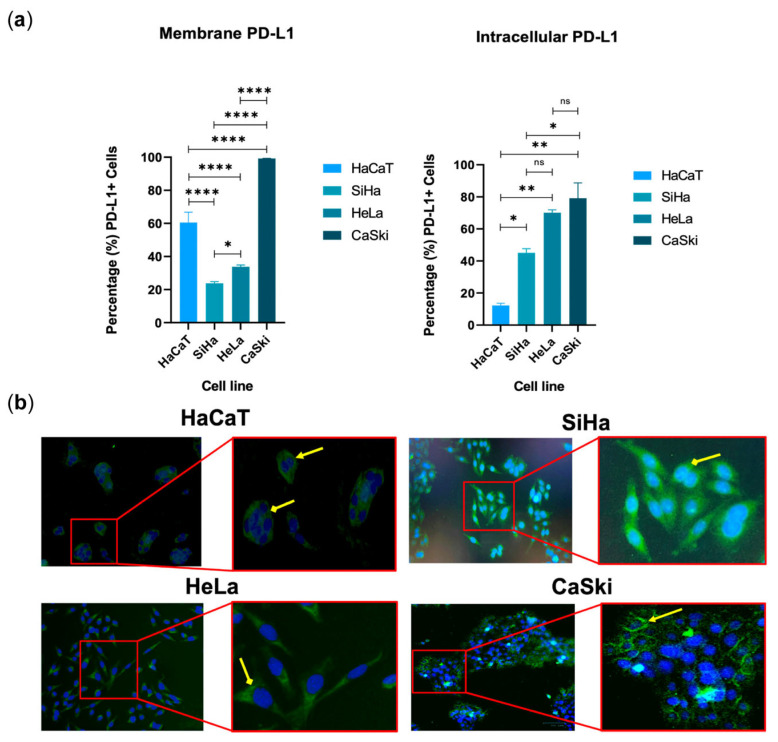
PD-L1 is present in the cell membranes and cytoplasm of cell lines derived from cervical cancer. (**a**) The percentages of PD-L1-positive cells were determined by traditional flow cytometry. Interestingly, the highest percentages of membrane PD-L1 were in CaSki cells. Data are shown as mean ± SD; two independent experiments were performed for each condition. * *p* ≤ 0.05, ** *p* ≤ 0.01, **** *p* ≤ 0.0001, ns: no significance. (**b**) Immunofluorescence was used to verify if PD-L1 (AF-488, green stain) was found in different subcellular locations, such as intracellular (cytoplasm marked with diamond-tipped arrow) and plasma membrane (closed arrow). Nuclei were stained with DAPI (blue). Images taken using the 10× objective (left side) and 30× (right side).

Continuing in the search for the cellular localization of the CMTM6/PD-L1 axis, we decided to measure the percentage of intracellular and plasma membrane-bound PD-L1 in cells derived from cervical cancer. We used SiHa, HeLa, and CaSki cells, with HaCaT cells (an immortalized human keratinocyte line) as a control (Figure 5). We found that PD-L1 was present intracellularly and in the plasma membranes of all cell lines, particularly in CaSki cells (remember that this is a cell line derived from metastasis). SiHa and HeLa cells exhibited the lowest percentages of membrane PD-L1, which coincides with the fact that these cell lines also had very low percentages of membrane CMTM6; likewise, CaSki cells had the highest levels of membrane PD-L1 (Figure 5a,b) and CMTM6 (Figure 4a), which would support the idea that both proteins are co-localized in the membrane. To determine if PD-L1 is also confined to other sub-cellular locations, we performed intracellular flow cytometry (Figure 5a) and immunofluorescence (Figure 5b), determining that this immunoligand is also found intracellularly. Interestingly, the intracellular signal was stronger than the membrane signal in the case of SiHa and HeLa cells, but not CaSki and HaCaT cells (Figure 5a), a phenomenon also observed above in the case of intracellular CMTM6 (which was relatively higher than the membrane signal in all four cell lines).

### 3.6. CMTM6 Released by Cervical Cancer-Derived Cell Lines

**Figure 6:**
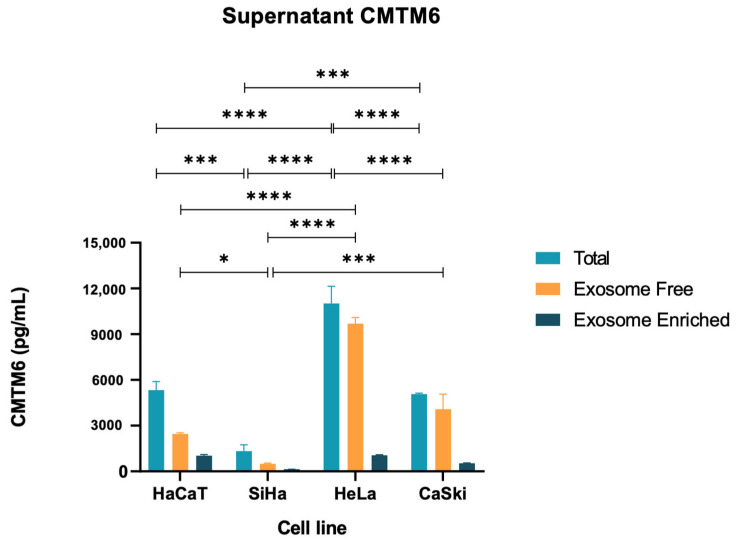
CMTM6 released by cell lines derived from cervical cancer. ELISA of culture supernatants to quantify the total levels of supernatant CMTM6. Detectable levels of supernatant CMTM6 were seen in all cell lines; however, HeLa cells were found to release the highest concentration (11,015 pg/mL). Data are shown as mean ± SD; two independent experiments were performed for each condition. * *p* < 0.05; *** *p* < 0.001; **** *p* < 0.0001.

To clarify if cervical cancer-derived cell lines also release supernatant CMTM6, we performed an ELISA of the cell culture supernatants of the four cell lines (SiHa, HeLa, CaSki, and HaCaT) (Figure 6). CMTM6 was quantified in the supernatant of the four cell lines, and HeLa cells were found to release the highest concentrations in both total and exosome-free experiments (Figure 6).

## 4. Discussion

The intrinsic relationship between PD-L1 and CMTM6 was recently described [18]. The membrane co-localization of both proteins has led to the proposal that CMTM6 is one of the main regulators of PD-L1, suggesting a potential value of CMTM6 as a therapeutic target to improve the efficacy of current anti-PD-L1 blocking therapies.

CMTM6 has been shown to bind to PD-L1 and thereby increase its half-life, presumably by preventing ubiquitination and lysosomal degradation during protein recycling. When CMTM6 is silenced, membrane PD-L1 expression decreases, demonstrating that CMTM6 is a positive regulator of PD-L1 expression [19]. Unfortunately, in cancer, the overexpression of PD-L1 has often been associated with immunoevasion and a poor prognosis for patients; while little is known about the role that CMTM6 found in novel non-canonical subcellular locations might play in cancer, the presence of this molecule in some cancers such as colorectal, melanoma, breast, lung, and pancreas has (most notably in pancreatic adenocarcinomas) been associated with shorter overall survival and enhanced prognostic value of PD-L1 expression, suggesting a cooperative effect between these two molecules in disease progression [20]. While CC is the second most frequent cause of cancer-related deaths in women, the involvement of CMTM6 in the context of CC is unclear [21]. It has been reported that high expression of CMTM6 is more common in tumor biopsy tissues from CC patients compared with adjacent normal tissues, and CMTM6 high vs. CMTM6 low tumor status was correlated with decreased patient overall survival time after surgery [22].

In the current study, we report, for the first time, the presence of soluble CMTM6 in cervical cancer. Expression of CMTM6 was higher in the plasma of CC patients compared to the plasma of HD controls. In fact, a literature search indicates that this is the first time, to the best of our knowledge, that increased CMTM6 in the plasma of patients with any cancer has been reported. The possible biological implications of CMTM6 in cancer have been little explored. High expression of CMTM6 in lung adenocarcinoma has been reported as a novel independent poor prognostic factor closely related to the tumor microenvironment [23]. Additionally, in an analysis of tumor biopsies, it was reported that CMTM6 gene expression was upregulated in numerous tumors, such as colon adenocarcinoma, glioblastoma multiforme, acute myeloid leukemia, lower-grade brain glioma, ovarian serous cystadenocarcinoma, pancreatic, rectal and stomach adenocarcinomas, thyroid carcinoma, and uterine corpus endometrial carcinoma. Gene Set Enrichment Analysis revealed the function of CMTM6 to be enriched in immune-response-related pathways such as natural-killer-cell-mediated cytotoxicity, chemokine signaling pathway, B cell receptor signaling pathway, and T cell receptor signaling pathway [24]. Given that CMTM6 is associated with many pathways, it is interesting to speculate that soluble CMTM6 may have similar interactions as well.

We also report that the expression of soluble PD-L1 was also higher in patients vs. controls. Oh et al. (2021), in a combined analysis of patients with different cancers, reported mean pre-checkpoint therapy plasma PD-L1 levels of 13.5 ± 12.1 pg/μL vs. 10.6 pg/uL in healthy donors. (Note that we reported a mean of 18.80 pg/mL in controls and 31.93 pg/mL in CC patients; while our HD values are higher than the Oh et al. paper, this can perhaps be explained by the use of different versions of the ELISA kit—the BMS 2212 kit used in the 2021 paper has been superseded by the now-validated kit for use with human samples (BMS2327) that we used). These authors suggest that high serum PD-L1 concentrations in patients with cancer may predict a low rate of disease control, and that increased levels are associated with worse survival in patients with advanced solid tumors (e.g., lung cancer, gastric cancer, renal cell carcinoma, melanoma, hepatocellular carcinoma, pancreatic cancer, and soft tissue sarcoma) [25]. Given that the magnitude of the change between HD and CC is greater in our cohort with cervical cancer, this suggests that serum PD-L1 may be an as-of-yet unexplored predictive or prognostic factor in patients with cervical cancer.

As we demonstrated, the soluble form of PD-L1 can be separated into exosome-free and exosome-enriched fractions. It is possible that the exosome-enriched form plays a critical role in the immune response. Exosome-associated PD-L1 was found to interact with T cell PD-1 and to induce immunosuppression of those T cells [26]. In fact, a report on patients with metastatic melanoma states that exosomal PD-L1 (and not just total soluble PD-L1, as mentioned above by Oh et al.) predicts treatment response and increases in line with IFN-γ; this is to say that increased IFN-γ (perhaps via chronic activation in the tumor microenvironment) increases exosomal PD-L1, leading to local and systemic suppression of the immune system and increased tumor growth. In line with this, patients with high pre-treatment exosomal PD-L1 were more likely to be non-responders, suggesting that pre-treatment exosomal PD-L1 might be a surrogate marker for the degree of PD1/PD-L1 mediated immune exhaustion. However, patients who showed the highest fold-change (greater than 2.43x) increase in exosomal PD-L1 in post- vs. pre-treatment were most likely to respond to treatment; the authors proposed that the increase in exosomal PD-L1 reflected an increase in antitumor immunity induced by the anti-PD1 checkpoint blockade [27]. Thus, the evaluation of exosomal PD-L1 in cervical cancer before and after checkpoint blockade will be an important next step for future studies.

When we fractioned the plasma from HDs and CC patients, we observed that both CMTM6 and PD-L1 were mainly confined to the exosome fraction, and that levels of CMTM6 and PD-L1 in patient exosomes were positively correlated. As mentioned above, PD-L1 has been reported in exosomes in other cancers and currently is an area of substantial investigation [27,28]. At present, only one report exists with respect to the presence of CMTM6 in exosomes; in this report, it was determined that tumor-cell-secreted exosomal CMTM6 induced M2-like macrophage polarization and contributed to malignant progression in oral squamous cell carcinoma [29]. The role or roles of exosome-associated CMTM6 in other cancers and with respect to exhaustion in cytotoxic cell populations or response to immune checkpoint blockade have yet to be explored.

In the analysis between the different cell lines, the highest expression of total and membrane PD-L1 was correlated with the highest expression of total and membrane CMTM6, both in CaSki cells, a model of metastatic cervical cancer. We have previously reported that plasma PD-L1 is positively correlated with PD-1+ T cells in women with low-grade lesions [7]; however, it remains to be seen if plasma CMTM6 can be correlated with tumor stage or with PD-1+ or exhausted T or NK cells, either in the tumor or in circulation. Here, we did not find any significant correlation between patient plasma CMTM6, either in total or exosomal form, and tumor stage. This may be due to the size of our study population, as we only had six samples in the low-grade group with exosomal CMTM6 results.

To try to further elucidate the non-canonical subcellular localizations, we also reported the presence of CMTM6 not just on the cell membranes of the four cell lines, but also in a secreted form present intracellularly, principally in the cytoplasm. Using cytometry, it was possible to report a faint membrane signal in SiHa, Hela, and HaCaT cells (1.40%, 3.78%, and 1.40%, respectively). This signal was effectively invisible when performing the immunofluorescence experiments. CaSki cells, in contrast, exhibited strong membrane CMTM6 staining with both immunofluorescence and cytometry. Intracellular cytometry staining showed SiHa, HeLa, and HaCaT cells to express notably less intracellular CMTM6 compared with CaSki cells, a result that was mirrored by the observations in the immunofluorescence experiments as well. HaCaT cells, the model of non-tumor keratinocytes, notably expressed the lowest values of membrane and intracellular CMTM6 in both sets of experiments. The predominance of the CaSki cell signaling for both CMTM6 and PD-L1 staining in both intracellular and membrane experiments reinforces the results seen in our Western blot experiments. With respect to the immunofluorescence staining, while it appeared that there might have been some nuclear and perinuclear CMTM6 staining as well, this will have to be correlated with confocal microscopy. Interestingly, CMTM6 has been found to stabilize nuclear P21 leading to inhibited tumor growth [30]. Previous reports have shown that CMTM6 molecules are located mainly on the cell membrane and in the cytoplasm [9], while the related molecule, CMTM4, is observed on the cell membrane, in the cytoplasm, and in the nucleus [31]. It is not clear what role the different stores of CMTM6 play. One clue might lie in the observation that HeLa cells, which exhibited a relatively high level of total cell lysate CMTM6, almost as high as CaSki cells, nonetheless were strikingly low in both membrane and intracellular CMTM6, but then demonstrated the highest levels of supernatant CMTM6 (most notably in total supernatant and the exosome-free fraction). It may be that certain intracellular stores of CMTM6 are more likely to be secreted.

Likewise, a number of groups have reported that PD-L1 may be secreted at different concentrations in different cell lines and patients with cancer; and some groups have found exosomal PD-L1 in cell lines of melanoma, breast, and lung cancers [27,32]. While other groups have reported this in cancer [28,33], we are the first to report the preferential expression of PD-L1 in exosomes vs. exosome-free fractions from patients with cervical cancer. In the future, it will be important to continue investigating the expression of exosomal PD-L1 in both cervical cancer patients (before and after treatment) and cervical cancer-derived cell lines.

It remains to be seen if cells with a low total CMTM6, and lower membrane expression but high secretion, exhibit a differential capacity to induce PD-L1 stability or induce exhaustion in cytotoxic cells (like NK or T cells) when compared to other cells (such as CaSki) with high total CMTM6 and high membrane expression but low total supernatant and low exosome CMTM6 secretion. Understanding this interplay may be important to clarify what possible additional biological roles CMTM6 is exerting beyond just being a PD-L1 stabilizer in the cell membrane and may open the door to investigating exosomal CMTM6 as a therapeutic target or prognostic marker.

## 5. Conclusions

In this work, we report that increased levels of soluble CMTM6 and PD-L1 were found in the plasma of patients with cervical cancer compared to a group of healthy donors. We report for the first time that CMTM6 is found in exosomes in cervical cancer patients, and that the exosome fraction of plasma exhibits higher levels of CMTM6 and PD-L1 concentrations compared to the exosome-free fraction.

In cervical cancer patients and cervical cancer-derived cell lines, we found that the expression of CMTM6 and PD-L1 are positively correlated; both patients and cell lines with higher levels of CMTM6 showed higher levels of PD-L1. The interesting question of the role of exosomal CMTM6 in antitumor immunity remains to be explored.

We identified CMTM6 and PD-L1 in the total lysate of cells derived from cervical cancer, i.e., SiHa, HeLa, and CaSki. In the search for non-canonical subcellular locations of CMTM6, we found it to be secreted in supernatant form and to be present both on the cell membrane and intracellularly.

## Figures and Tables

**Table 1:** Description of the cohort subjects (CMTM6).

	Healthy Donors	Cervical Cancer Patients
	(*n* = 23)	(*n* = 23)
**Age**		
Mean in years (range)	45 (25–79)	46 (25–72)
**Histopathology**		
Squamous cell carcinoma	-	12 (52%)
Adenocarcinoma	-	1 (4%)
No information	-	10 (44%)
**FIGO Stage**		
I		3 (13%)
II		3 (13%)
III		8 (35%)
IV		5 (22%)
No information		4 (17%)
**Treatment scheme status**		
Pre-treatment	-	22 (96%)
Post-treatment	-	1 (4%)
Chemotherapy	-	1 (4%)
Chemotherapy + Radiotherapy	-	0 (0%)
Disease free survival	-	0 (0%)
No information		0 (0%)

**Table 2:** Description of the cohort subjects (PD-L1).

	Healthy Donors	Cervical Cancer Patients
	(*n* = 11)	(*n* = 21)
**Age**		
Mean in years (range)	43 (25–63)	44 (25–79)
**Histopathology**		
Squamous cell carcinoma	-	13 (65%)
Adenocarcinoma	-	0 (0%)
No information	-	7 (35%)
**FIGO Stage**		
I		3 (14%)
II		2 (10%)
III		7 (33%)
IV		5 (24%)
No information		4 (19%)
**Treatment scheme status**		
Pre-treatment	-	16 (76%)
Post-treatment	-	5 (24%)
Chemotherapy	-	1 (5%)
Chemotherapy + Radiotherapy	-	1 (5%)
Disease free survival	-	3 (14%)
No information	-	0 (0%)

## Data Availability

Data are contained within the article.

## Supplementary Materials

The following supporting information can be downloaded at: https://www.mdpi.com/article/10.3390/cancers16183126/s1, Figure S1. Uncropped western blot figures of Figure 2e; Figure S2. Uncropped western blot figures of Figure 3a,b.
