# Supplementary material for: New Advances in the Study of CMTM6, a Focus on Its Novel Non-Canonical Cellular Locations, and Functions beyond Its Role as a PD-L1 Stabilizer
Source: Cancers (Basel). 2024 Sep 11;16(18):3126. doi: 10.3390/cancers16183126 (PMC11430317; doi:10.3390/cancers16183126)
Supplement: Supplementary file 1 [file cancers-16-03126-s001.zip › cancers-3188131-supplementary.pdf]

Supplementary Figure

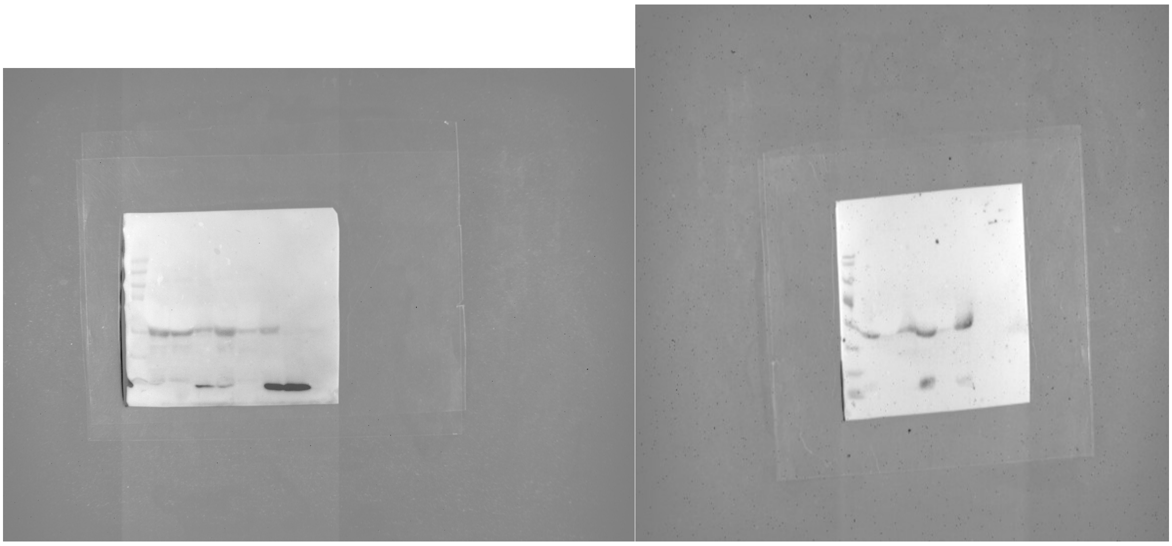

Figure S1. Uncropped western blot figures of figure 2 (e).

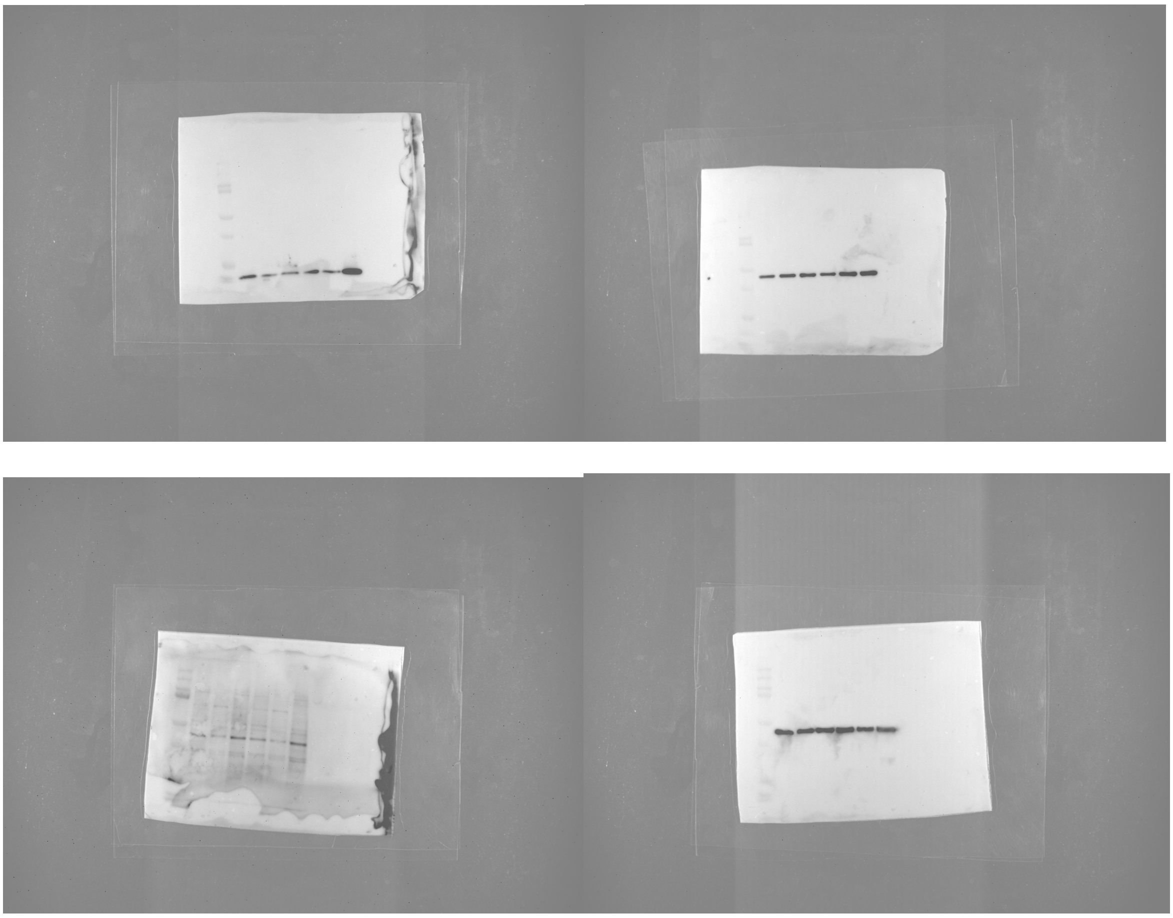

Figure S2. Uncropped western blot figures of figure 3 (a) and figure 3 (b)
